# Ischemia-Reperfusion Injury and Pregnancy Initiate Time-Dependent and Robust Signs of Up-Regulation of Cardiac Progenitor Cells

**DOI:** 10.1371/journal.pone.0036804

**Published:** 2012-05-09

**Authors:** Rami Genead, Helene Fischer, Alamdar Hussain, Marie Jaksch, Agneta B. Andersson, Karin Ljung, Ivana Bulatovic, Anders Franco-Cereceda, Elzafir Elsheikh, Matthias Corbascio, C. I. Edvard Smith, Christer Sylvén, Karl-Henrik Grinnemo

**Affiliations:** 1 Division of Cardiology, Department of Medicine, Karolinska Institutet, Stockholm, Sweden; 2 Department of Physiology, Karolinska Institutet, Stockholm, Sweden; 3 Department of Laboratory Medicine, Clinical Research Center, Karolinska Institutet, Stockholm, Sweden; 4 Department of Biosciences, COMSATS Institute of Information Technology, Chak Shahzad Campus, Islamabad, Pakistan; 5 Division of Cardiothoracic Surgery and Anaesthesiology, Department of Molecular Medicine and Surgery, Karolinska Institutet, Karolinska University Hospital, Stockholm, Sweden; University of Frankfurt - University Hospital Frankfurt, Germany

## Abstract

To explore how cardiac regeneration and cell turnover adapts to disease, different forms of stress were studied for their effects on the cardiac progenitor cell markers c-Kit and Isl1, the early cardiomyocyte marker Nkx2.5, and mast cells. Adult female rats were examined during pregnancy, after myocardial infarction and ischemia-reperfusion injury with/out insulin like growth factor-1(IGF-1) and hepatocyte growth factor (HGF). Different cardiac sub-domains were analyzed at one and two weeks post-intervention, both at the mRNA and protein levels. While pregnancy and myocardial infarction up-regulated Nkx2.5 and c-Kit (adjusted for mast cell activation), ischemia-reperfusion injury induced the strongest up-regulation which occurred globally throughout the entire heart and not just around the site of injury. This response seems to be partly mediated by increased endogenous production of IGF-1 and HGF. Contrary to c-Kit, Isl1 was not up-regulated by pregnancy or myocardial infarction while ischemia-reperfusion injury induced not a global but a focal up-regulation in the outflow tract and also in the peri-ischemic region, correlating with the up-regulation of endogenous IGF-1. The addition of IGF-1 and HGF did boost the endogenous expression of IGF and HGF correlating to focal up-regulation of Isl1. c-Kit expression was not further influenced by the exogenous growth factors. This indicates that there is a spatial mismatch between on one hand c-Kit and Nkx2.5 expression and on the other hand Isl1 expression. In conclusion, ischemia-reperfusion injury was the strongest stimulus with both global and focal cardiomyocyte progenitor cell marker up-regulations, correlating to the endogenous up-regulation of the growth factors IGF-1 and HGF. Also pregnancy induced a general up-regulation of c-Kit and early Nkx2.5+ cardiomyocytes throughout the heart. Utilization of these pathways could provide new strategies for the treatment of cardiac disease.

## Introduction

The adult heart is not a terminally differentiated organ but maintains regenerative capacity through out life [1]. During the cold war nuclear bomb tests released carbon-14 (^14^C) into the atmosphere, which was then incorporated into the genomic DNA of proliferating cells. Because of this phenomenon it is now possible for the first time to determine the age of cardiomyocytes in the adult heart. If one discounts the polyploidisation of DNA in cardiomyocytes during childhood [2], [3], then there is a turn over of 1% of cardiomyocytes per year at the age of 25 and 0.45% of cardiomyocytes at the age of 75. This implies that 50% of our cardiomyocytes have been replaced during our lifetime.

The concept of myocardial regeneration by means of stimulating or augmenting the endogenous regenerative potential in situ is an attractive approach. This offers distinct advantages to stem cell implantation since the problems with engraftment and immune rejection are avoided. Despite of the recent enthusiasm, several issues remain unsolved, like which progenitor cells are responsible for the normal myocardial homeostasis and which stem cells are up-regulated most during a response to physiological and pathological stress? Many research groups have reported on different types of stem cell- like cells from different species. These include cells with surface expression of molecules like stem cell antigen-1 (Sca-1) [4], [5], [6], Abcg2 [7], [8], [9], c-Kit [10], [11], [12], [13], [14], [15] and the transcription factors Tbx 5 and Islet-1 (Isl1) [16], [17], [18], [19]. It is still unclear if progenitor cells which express Sca-1, c-Kit or Abcg2 all come from the same stem cell and represent different physiological states or if they are different cell types [20]. Of the described cells, only the cells expressing c-Kit demonstrate the stem cell characteristics like clonogenicity, self-renewal and multipotency [10]. Miyamoto and co-workers have isolated and successfully cultured c-Kit+ cells from adult rat hearts for 40 passages [13]. These cells maintained their stem cell characteristics and could differentiate into cardiomyocytes, smooth muscle and endothelial cells. Both side population cells expressing Abcg2 and some Sca-1+ cells, co-express c-Kit [4], [7], [8], [9], which implies that these cells are derived from multipotent c-Kit+ cells. Goumans et al. have presented results where cardiomyocyte progenitor cells have been isolated from human fetal and adult hearts using a mouse antibody for Sca-1 [21]. The isolated cardiomyocyte progenitor cells expressed moderate levels of c-Kit and could differentiate into cardiomyocytes after stimulation with 5′-azacytidine and TGF-β1.This finding further emphasizes the concept that c-Kit+ cells can be cardiomyocyte progenitor cells both in rodents and in man.

Other important cardiovascular progenitors are the Isl1+ cells, which form the second heart field during organogenesis [16]. In vivo cell lineage tracing in mouse embryos using the cre-loxP strategy has confirmed that Isl1+ progenitors contribute to more than two-thirds of the cells in the embryonic heart [18], [22], [23]. The majority of Isl1+ cells in rat embryos are localized to the outflow tract and differentiate to cardiomyocytes where they express both troponin T (TnT) and α-smooth muscle actin (α-SMA) [17]. Isl1+ cells have also been localized to the outflow tract of adult pregnant rat hearts [17], which could imply that they contribute to the generation of cardiomyocytes during stress.

Dual delivery of insulin like growth factor-1 (IGF-1) and hepatocyte growth factor (HGF) from affinity-bound biomaterial has previously shown to reduce apoptosis, induce cardiomyocyte cell-cycle re-entry and increase the incidence of GATA-4 positive cell clusters in a rat myocardial infarction model [24].Therefore it is interesting to study if endogenous IGF-1 and HGF are involved in the up-regulation of cardiac progenitors due to stress and if local administration of these growth factors could further potentiate this effect.

Subsequently there seems to be at least two cardiac progenitor cells; one which expresses c-Kit, and one which expresses Isl1. This is the first blinded longitudinal study, which explores the up-regulation of cardiac progenitor cells during different forms of physiological and pathological stress like pregnancy, myocardial infarction, and ischemia-reperfusion injury. We believe that this study indicates that there exists a cardiogenic c-Kit+ population and that these progenitor cells together with Isl1+ cells take part in myocardial regeneration following pathological and physiological stress.

## Materials and Methods

### Experimental Design

**Figure 1:**
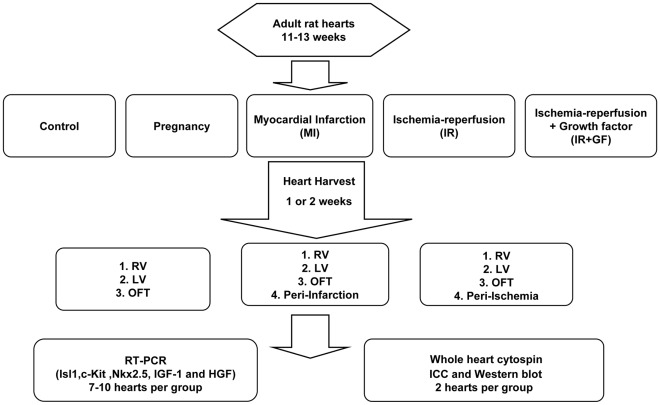
Flow chart showing the experimental study design. In figure; RV: Right ventricle, LV: Left ventricle, OFT: Outflow tract, RT-PCR: Real Time Polymerase Chain Reaction and ICC: Immunocytochemistry.

The study design is shown in Figure 1. All the procedures were approved (approval ID S68-09 and S175-09) by the Animal Care Committee of Karolinska University Hospital, Stockholm, Sweden and conform to the Guide for the Care and Use of Laboratory Animals published by the US National Institutes of Health *(NIH Publication No.85-23, revised 1996*). The rats were allowed free access to water and conventional pellet diet.

In this study we used female adult (11–13 weeks old) Sprague Dawley rats (Charles River, Germany) which were divided into five groups; control, pregnancy, myocardial infarction and ischemia-reperfusion with and without growth factors. The control rats (n = 13) were not subjected to any surgical event. The pregnant rats (n = 10) used were sacrificed on gestational day (GD) 13, since we have previously shown presence of Isl1+ cells in the outflow tract at this time of pregnancy [17]. The rats from the myocardial infarction group (MI, n = 15), ischemia-reperfusion groups (IR) without (IR, n = 17) or with (IR+GF, n = 16) the growth factors HGF, IGF-1, were euthanized at the two time points; 1 week and 2 weeks. At the time of harvest, the hearts were divided into the different regions; outflow tract (OFT, defined as the outflow tract of the right ventricle), right ventricle (RV), left ventricle (LV), area of peri-infarct and peri-ischemia. Subsequent analysis was performed by Real Time Polymerase Chain Reaction (RT-PCR), Western blot, and immunocytochemistry (ICC), in a blinded manner.

### Anesthesia and Postoperative Care

The rats that underwent myocardial infarction or ischemia-reperfusion injury were generally anesthetized with a subcutaneous injection of Midazolam (Dormicum, 5 mg/kg) (Algol Pharma AB, Germany), Medetomidin hydrochloride (Domitor vet, 0.1 mg/kg) (Orion Corp., Espoo, Finland), Fentanyl (0.3 mg/kg) (B.Braun Medical AG, Seesatz, Switzerland) and subsequently endotracheally intubated. Positive-pressure ventilation was kept at a rate of 100 cycles per minute with a tidal volume of 4–5 ml with room air using a ventilator (7025 Rodent ventilator, UGO BASILE S.R.L, Italy).

The anesthesia was reversed by an intramuscular injection of Flumazenil (Lanexat, 0.1 mg/kg) (Hameln Pharma, Germany) and Tipamezol hydrochloride (Antisedan vet 5 mg/kg) (Orion Corp., Espoo, Finland). Postoperative analgesia was maintained by administrating Buprenorphin hydrochloride (Temgesic, 0.004 mg/kg/twice per day for 3 days) (Schering-Plough Corp., Belgium). Rats that showed signs of malfunction were excluded from the study.

### Induction of Myocardial Infarction and Ischemia-reperfusion Injury

In the myocardial infarction and ischemia-reperfusion groups, the hearts were exposed through a left lateral thoracotomy. In the myocardial infarction group the left anterior descending artery (LAD) was permanently ligated and infarction induction was confirmed by color change and dyskinesia of the antero-lateral wall of the left ventricle. In the ischemia-reperfusion groups, the LAD was temporary ligated for 5 minutes. In the (IR+ GF) group this was followed by an intramyocardial injection of rrIGF-1(8 µg) and rhHGF (2 µg) (4326RG and 294HG/CF, R & D systems, Minneapolis, USA) dissolved in 10% rat serum in PBS and in the IR-group by an intramyocardial injection of corresponding volume of 10% rat serum.

### Sample Preparation and RNA Extraction

The hearts were harvested through a left thoracotomy. The hearts were kept cold on dry-ice and subsequently divided into the different subdomains; OFT, RV, LV and when applicable the area of peri-ischemia and peri-infarct. All the heart samples were snap-frozen in liquid nitrogen and kept in minus 70°C until RNA extraction. Total RNA was extracted from the heart samples according to the QuickGene RNA tissue assay (RT-S2, Science Imaging Scandinavia AB, Sweden) using the Fuji QuickGene-QG-810/QG-800Mini80 RNA isolation system. An average of 15–30 mg heart tissue per each sample underwent a homogenization process in a homogenization tube containing lysis buffer and 0.5 mm zirconium oxide using the Bullet Blender Homogenizer ™ (Next Advance Inc., USA). The eluted samples were concentrated to an average of 200 ng RNA/µl using the speed vacuum system (Max Dry Lyo machine).

The RNA concentrations and purities (A260/280) were measured in a NanoDrop spectrophotometer® (ND-1000) (Nanodrop technologies, Wilmington, DE, USA). The RNA quality was further evaluated by 1% agarose-gel electrophoresis. These measurements were performed before and after the concentration step to assure proper selection of the high quality samples.

### Quantitative Real-Time Polymerase Chain Reaction (RT-PCR)

Two micrograms of total RNA was reverse transcribed by Superscript reverse transcriptase (Life Technologies, Stockholm, Sweden) using random hexamer primers (Roche Diagnostics GmbH, Mannheim, Germany) in a total volume of 20 µl.

Real-time PCR was used to measure mRNA expression on a 7500 Fast real-time PCR system (Applied Biosystems Inc., Foster City, CA, USA). Primers and probes were supplied as a TaqMan® Reagents kit (Applied Biosystems), Isl1 Rn00569203 m1, Kit Rn 00573942 m1, Nkx 2.5 Rn 00586428 m1, IGF-1 Rn 00710306 m1, HGF Rn 00566673 m1, Gapdh Rn 00000016 m1. GAPDH was used as an endogenous control to correct for potential variation in cDNA loading.

All PCR reactions were performed in 96-well MicroAmp Optical plates (Applied Biosystems). cDNA was diluted 1∶5. Amplification reagents (10 µl) contained 1 µl sample for c-Kit and Nkx 2.5 and 5 µl for Isl1 in TaqMan Universal PCR Mastermix.

All samples were run in duplicates. Each RT-PCR cycle consisted of: Initial activation stage at 95°C (10 min), then the denaturation stage at 95°C (15 sec) and the annealing stage at 60°C (60 sec) cycled 40 times.

The comparative ΔCt method was used to calculate the relative gene expression to GAPDH for the genes analyzed [25].

### Immunocytochemistry

The whole hearts were mechanically minced into small pieces, treated with collagenase type II solution (CLS-2, Worthington, Biochemical Corporation, USA) to receive single cell suspensions for subsequent cytospin. To identify mast cells in the cytospin preparations, we used Toluidine blue staining [26]. Each cytospin was fixed in 4% formalin, washed in dH_2_O and immersed in 1% Toluidineblue (Toluidineblue, 1B-481, Waldeck, Germany) in 70% ethanol, pH 1.5 for 10 seconds and visualized under light microscope. Mast cells stain red-purple (metachromatic staining) and the background stain blue (orthochromatic staining). Semi-quantitative analyses to determine the relative number of mast cells to the total number of cells in the cytospins were performed. Four whole heart cytospins from two rats per group were counted in the light microscope.

### Immunofluorescence Staining

The adult rat hearts from each group were freeze-sectioned into 5-µm-thick sections, except for the Isl1 cell detection where whole heart cytospins were prepared. The frozen sections or cytospined cells were fixed in 4% formaldehyde, ice-cold methanol, or acetone, blocked with serum followed by incubation with the primary antibodies; mouse anti-rat supernatant Isl1 (1∶800, DSH 39.4 D5-s; Columbia University, USA), human NKx2.5 monoclonal mouse IgG1 antibody (1∶75, R&D MAB2444, clone 259416, Minneapolis, MN) and mouse SCF R/c-Kit goat polyclonal IgG antibody (1∶40, R&D, AF1356). In the next step, the sections or cells were incubated with the fluorescence-labeled secondary antibodies; AlexaFluor 488 for c-Kit and Isl1 and AlexaFluor 546 for Nkx2.5 (Invitrogen, Carlsbad, CA) and visualized in the fluorescence microscope (Olympus BX60; Olympus Optical Co. Ltd., Tokyo, Japan) using diamidino-2-phenylindole (DAPI) in the mounting medium. As positive controls we used human fetal hearts for Nkx2.5; human fetal hearts, rat embryos, and an insulin cell line (INS 1E) for Isl1 and for c-Kit we used adult rat spleen, adult rat kidney and rat embryos. As negative controls, the primary antibody was omitted and staining was only performed with the secondary antibody. To test for autofluorescence, staining was performed without both primary and secondary antibodies.

Semi-quantitative analyses were performed to determine the relative number of cells expressing the different markers (Nkx2.5, c-Kit and Isl1) in relation to the total number of cells per each field. Slides (n = 3–4 from each heart, 2 hearts from each group) were counted at 40 x magnification in the fluorescence microscope. Due to the scarcity of detected Isl1 positive cells, semi-quantitative analysis was performed on cells expressing Nkx2.5 as well as c-Kit.

### Western Blot

Cells from cytospins (3–4 slides/group) were directly lysed with 100 µL Laemmli sample buffer and proteins were separated on gradient 4–12% Bis-Tris NuPAGE gels. Proteins were transferred to nitrocellulose membranes using Iblot (Invitrogen, USA) and total protein loadings were assessed with Ponceau S solution (P7170-1L, Sigma Life Science, Sweden). Membranes were blocked with non-fat 5% dry milk for one hour in room temperature (RT) and were incubated over-night (4°C) with mouse monoclonal c-Kit (MS-289-P1, Thermo scientific, UK) 1∶1000, mouse anti rat supernatant Isl1 (DSH 39.4 D5-s, Colombia University, USA) 1∶500 and polyclonal goat Nkx2.5 (SAB2501264, Sigma-Aldrich, Sweden) 1∶1000. GAPDH was used as a loading control; mouse monoclonal GAPDH (ab8245, Abcam, UK) 1∶5000. Membranes were washed four times with PBST and incubated with the secondary antibodies (Li-COR goat anti-mouse 926-32210 or 926-6820 and donkey anti-goat 926-32214) 1∶20 000, for one hour in RT. Membranes were washed three times with PBST then twice with PBS and finally scanned using Li-COR scanner. Experiments were run in triplicates.

### Statistics

All data are expressed as mean ± standard deviation. Non-parametric (Mann-Whitney U) test was employed to calculate the statistical significance between two independent groups. Statistical correlations were analyzed with the Pearson correlation test.

A *P<0.05* and *P<0.001* were considered to be statistically significant and highly significant respectively. Statistical analysis was performed with the SPSS software version 17.0.

## Results

### The Effect of Ischemia-reperfusion Injury, Myocardial Infarction and Pregnancy on the Expression of c-Kit, Isl1 and Nkx2.5

**Figure 2:**
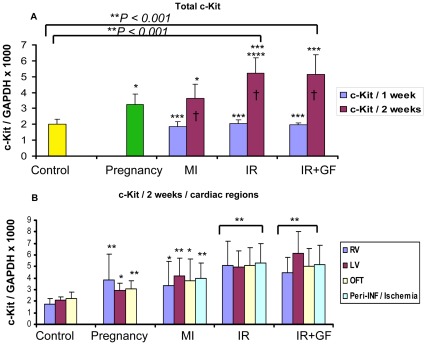
Quantitative RT-PCR showing the time-dependent distribution of c-Kit mRNA in the different groups. The c-Kit mRNA expression in the different groups is related to the expression of GAPDH. Fig A shows the mean of the whole heart c-Kit mRNA expression in each group. In fig B, the distribution of the c-Kit mRNA between each region at 2 weeks is demonstrated. Number of animals is 7–10 per group (see flow chart in Fig 1). In figure; MI: myocardial Infarction; IR: ischemia-reperfusion; IR+GF: ischemia-reperfusion+ growth factors. RV: Right ventricle, LV: Left ventricle, OFT: Outflow tract and Peri-INF/Ischemia: Peri-infarction and Ischemia region respectively. Data is presented as mean ± SD. *P<0.05 vs. Control group, ** *P<*0.001 vs. Control group, ***P<0.05 vs. pregnancy group, ****P<0.05 vs. MI (2 weeks) group and † *P<0.05* 1 week vs. 2 weeks. In fig B, each region is related to corresponding region of the control, pregnancy, myocardial infarction and ischemia-reperfusion w/o growth factors.

**Figure 3:**
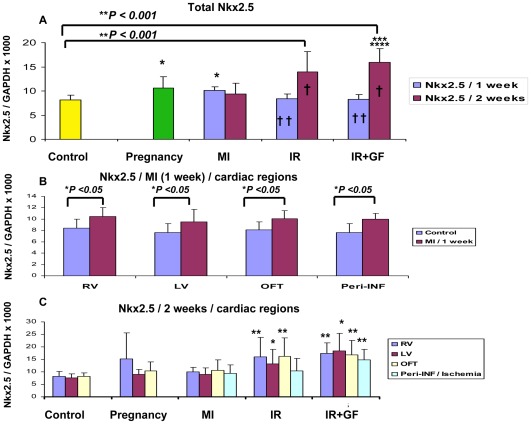
Quantitative RT-PCR showing the time-dependent distribution of the Nkx2.5 early cardiomyocyte progenitor in the different groups. The Nkx2.5 mRNA expression in the different groups was related to the expression of GAPDH. Fig A shows the mean of the whole heart Nkx2.5 mRNA expression in each group. In fig B, the distribution of Nkx2.5 mRNA between each region of the myocardial infarction group at 1 week is demonstrated. In fig C, the Nkx2.5 mRNA distribution between each region is demonstrated at 2 weeks. Number of animals is 7–10 per group (see flow chart in Fig 1). In figure; MI: myocardial infarction; IR: ischemia-reperfusion; IR+GF: ischemia-reperfusion+ growth factors. RV: Right ventricle, LV: Left ventricle, OFT: Outflow tract and Peri-INF/Ischemia: Peri-infarction and Ischemia region respectively. Data is presented as mean ± SD. *P<0.05 vs. Control group, ** *P*<0.001 vs. Control group, ***P<0.05 vs. pregnancy group, ****P<0.05 vs. MI (2 weeks) group and † *P*<0.05, 1 week vs. 2 weeks and †† *P*<0.05 vs. MI (1 week) group. In fig B and C the mRNA expression of each region is related to the corresponding region of the control group.

In this study we have chosen to utilize c-Kit and Isl1 as markers for cardiomyocyte progenitor cells and Nkx2.5 [27] as a marker to detect early differentiation into cardiomyocytes. Based on the quantitative RNA analysis from the different groups, ischemia-reperfusion injury (IR and IR+GF) induced a stronger up-regulation of c-Kit, Isl1 and Nkx2.5 versus the control group than both myocardial infarction and pregnancy (Fig 2A, 3A, 4A). In the ischemia-reperfusion groups, the up-regulation of c-Kit, Isl1 and Nkx2.5 was mainly seen after 2 weeks (Fig. 2A, 3A, 4A). After myocardial infarction (MI), there is an inverted trend between c-Kit and Nkx2.5 expression. The Nkx2.5 expression reached its maximum already at one week (Fig 3A), while for c-Kit this occurred at two weeks (Fig 2A).

**Figure 4:**
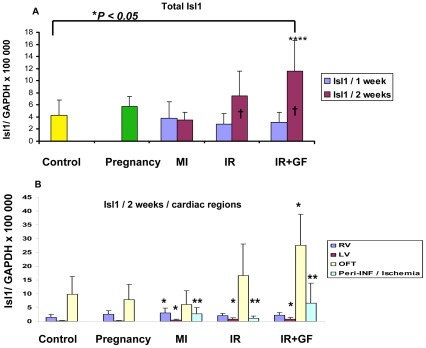
Quantitative RT-PCR showing the time-dependent distribution of Isl1 mRNA in the different groups. The Isl1 mRNA expression in the different groups was related to the expression of GAPDH. Fig A shows the mean of the whole heart Isl1 mRNA expression in each group. In fig B, the distribution of Isl1 mRNA between each region is demonstrated at 2 weeks. Number of animals is 7–10 per group (see flow chart in Fig 1). In figure; MI: myocardial infarction; IR: ischemia-reperfusion; IR+GF: ischemia-reperfusion+ growth factors. RV: Right ventricle, LV: Left ventricle, OFT: Outflow tract and Peri-INF/Ischemia: Peri-infarction and Ischemia region respectively. Data is presented as mean± SD. *P<0.05 vs. Control group, ***P<0.05 vs. pregnancy group, ****P<0.05 vs. MI (2 weeks) group and † *P*<0.05, 1 week vs. 2 weeks. In fig. B the mRNA expression of each region is related to the corresponding region of the control group.

Ischemia-reperfusion injury induced a similar up-regulation of c-Kit in all of the studied sub-domains; right ventricle, left ventricle, outflow tract area and area of peri-ischemia (Fig 2B). In myocardial infarction there was a general up-regulation of c-Kit, with a more robust c-Kit response in the remote left ventricle and the area of peri-infarction compared to the corresponding left ventricle in the control group (Fig 2B).

**Figure 5:**
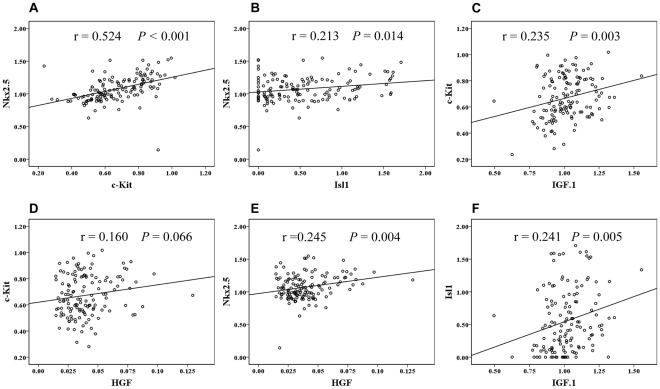
Correlations between cardiomyocyte progenitors and endogenous IGF-1/HGF mRNA expressions in the different groups. The scatter plots on logged data in Fig 5A, B and E reveal that Nkx2.5 is positively correlated with c-Kit, Isl1 and HGF expression. c-Kit is also positively correlated to IGF-1 expression (Fig 5C) and there is a borderline relationship to HGF expression (Fig 5D). As shown in fig F, Isl1 is positively correlated with IGF-1 expression. In figure; R = Pearson correlation coefficient.

There is a highly significant correlation between the up-regulation of c-Kit, which occurred two weeks after ischemia-reperfusion injury and the up-regulation of the early cardiomyocyte marker Nkx2.5 (*P*<0.001, r = 0.524) (Fig 3A and 5A). This up-regulation of Nkx2.5, which was induced by ischemia-reperfusion injury, took place globally and in the same cardiac sub-domains as the increased expression of c-Kit (Fig 3C). Unexpectedly, there was an inverted temporal response between c-Kit and Nkx2.5 expression following myocardial infarction, where c-Kit expression increased from week 1 to week 2 while Nkx2.5 expression reached its maximal level already after one week (Fig 2A, 3B). The reaction was not focal but global throughout the myocardium, with no differences between the different sub-domains of the heart (Fig 3B).

Initially the expression of Isl1 was very low, in the order of about 1/500 of the mRNA expressions of c-Kit and Nkx2.5. In contrary to the expression of these progenitor markers, the Isl1 was focally expressed, primarily localized to the outflow tract and the right ventricle (see control group Fig 4A, B). Ischemia-reperfusion injury induced a robust up-regulation of Isl1 expression compared to myocardial infarction (Fig 4A). The up-regulation induced by ischemia-reperfusion injury and myocardial infarction was mainly in other sub-domains than the up-regulation of c-Kit. In contrast to the c-Kit response, the up-regulation of Isl1 as a response to ischemia-reperfusion and myocardial infarction was mainly located in the outflow tract area, but also in the remote areas of the left ventricle and peri-ischemic regions (Fig 4B). Up-regulation of Isl1 correlated also to the Nkx2.5 response (*P* = 0.01, r = 0.213) (Fig 5B), even if this correlation was weaker than for c-Kit.

We also wanted to study if dual delivery of the growth factors HGF and IGF-1 could boost the effects induced by ischemia-reperfusion injury. IGF-1 and HGF did not affect the expression of c-Kit (Fig 2A, B). Interestingly, there was a trend where the expression of Nkx2.5 increased after treatment with the growth factors, primarily in the remote left ventricle and peri-ischemic regions (Fig 3C).The expression of Isl1 was also stimulated by IGF-1 and HGF, especially in the outflow tract area. Even if the expression of Isl1 in the peri-ischemic region was lower than that seen in the outflow tract, following ischemia-reperfusion injury with the addition of growth factors there was a substantial up-regulation of Isl1 in this area compared to the control group (Fig 4A, B).

Pregnancy, also to a certain degree, stimulated the up-regulation of c-Kit and Nkx2.5 (Fig 2A, 3A) to a level equivalent to the stimulation seen after myocardial infarction. The response was similar in all regions of the heart (Fig 2B, 3C). However, pregnancy did not seem to affect the expression of Isl1 (Fig 4A).

### Endogenous IGF-1 and HGF are Involved in the Up-regulation of Cardiac Progenitors

**Figure 6:**
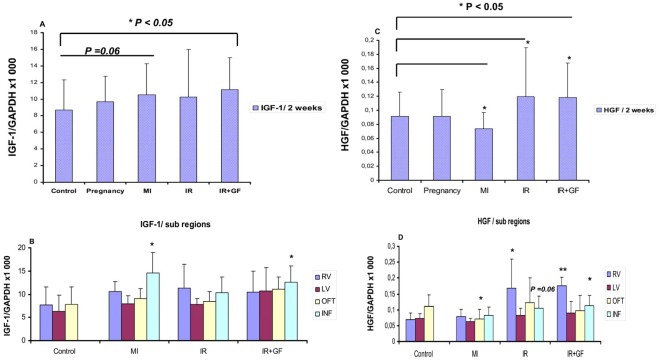
Quantitative RT-PCR showing the endogenous expression of IGF-1 and HGF mRNA at two weeks in the different groups. The IGF-1 and HGF mRNA expression is related to the expression of GAPDH. Fig A and C, show the mean whole heart expression of IGF-1 and HGF in the different groups, while in fig B and D the growth factor expression in each sub-domain is demonstrated. Number of animals is 7–10 per group (see flow chart in Fig 1). In figure; MI: myocardial Infarction; IR: ischemia-reperfusion; IR+GF: ischemia-reperfusion+ growth factors. RV: Right ventricle, LV: Left ventricle, OFT: Outflow tract and INF: Peri-infarction and Ischemia region respectively. Data is presented as mean± SD. *P<0.05 and **P<0.001 vs. Control group. In fig. C and D the mRNA expression of each region is related to the corresponding region of the control group.

The endogenous up-regulation of IGF-1 and HGF followed the same pattern as for cardiac progenitors in the ischemia-reperfusion groups w/o growth factors at two weeks (Fig 6A, C respectively). Interestingly, the sub-domain analysis revealed a preferential up-regulation of IGF-1 at the site of injury (*P*<0.05) (Fig 6B), while the HGF expression correlated with a more diffuse up-regulation in the peri-ischemic region as well as in the right ventricle (*P*<0.05 and *P*<0.001 respectively) (Fig 6D).

In the myocardial infarction group there was a divergent reactivity, with marginal up-regulation of IGF-1 (*P* = 0.06) (Fig 6A), especially at the site of injury (Fig 6C) and significant (*P*<0.05) down-regulation of endogenous HGF (Fig 6C and D).

The observed up-regulations of cardiac progenitors in the pregnancy group did not seem to be mediated by IGF-1 or HGF (Fig 6A, B).

To further investigate the role of IGF-1/HGF in the up-regulation of the different cardiac progenitor markers, correlation analyses were performed. According to these results, c-Kit mRNA expression was shown to correlate to the IGF-1/HGF expression (*P* = 0.003, r = 0.235, and *P* = 0.066, r = 0.160, respectively) (Fig 5C, D).

Furthermore, Nkx2.5 up-regulation was positively (*P* = 0.004, r = 0.245) correlated to endogenous HGF expression but not IGF-1 (Fig 5E), while Isl1 expression was related to (*P* = 0.005, r = 0.241) endogenous IGF-1 expression (Fig 5F).

### Correlation Between mRNA and Protein Expression

**Figure 7:**
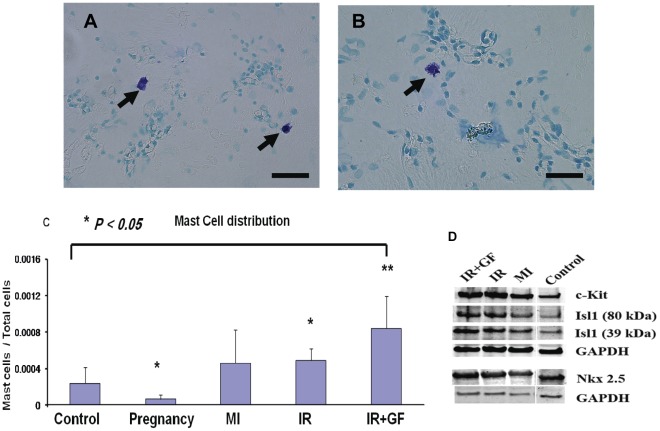
Mast cell distribution and Western blot analysis. Fig A-C: Two weeks after the intervention, mast cells were detected in whole heart cytospins from the different groups using toluidineblue staining. As seen in fig A and B, it is easy to detect the mast cells as red-purple cells. Fig C shows a semi-quantitative analysis of the distribution of mast cells among the different groups. Four whole heart cytospins from two rats per group were counted in the light microscope. Data is presented as mean± SD. *P<0.05 vs. Control group and **P<0.05 vs. MI (2 weeks) group. Fig D: The protein expression for c-Kit, Isl1 and Nkx2.5 is related to the GAPDH expression in each group at 2 weeks. The Isl1 protein expression showed a band at 39 kDa and another band was consistently observed at 80 kDa. Experiments were run in triplicates (n = 2 per group). In figure; MI: myocardial infarction; IR: ischemia-reperfusion; IR+GF: ischemia-reperfusion+ growth factors. Bars in panel (A) = 25 µm and in panel (B) = 50 µm.

The mRNA expression patterns of c-Kit, Isl1 and Nkx2.5 were confirmed at the protein level at two weeks using both immunohistochemistry (IHC) and Western blot analyses. The protein expression levels determined by Western blot correlated consistently with the mRNA data, with the highest protein expression of c-Kit, Isl1 and Nkx2.5 in the ischemia-reperfusion injury groups (IR and IR+GF) (Fig 7D **)**. The protein analysis in the pregnancy group was not reliable due to inter-individual variation of protein level differences. These variations might be due to post-translational modifications (data not shown).

**Figure 8:**
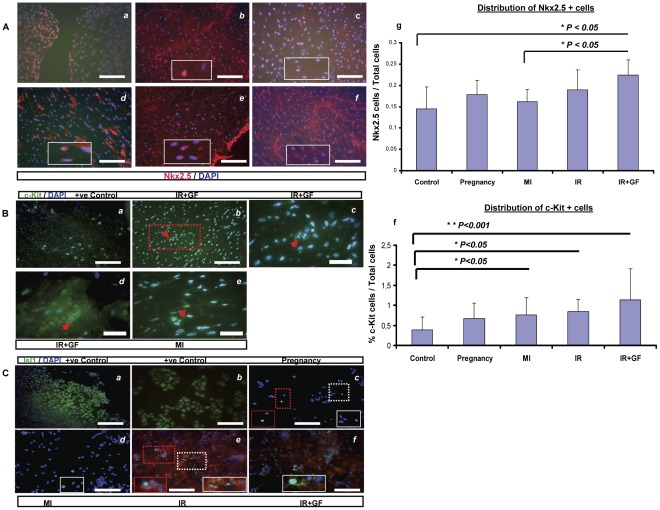
Immunohistochemical analysis of Nkx2.5, c-Kit and Isl1 cardiac progenitors. **Section A:** The distribution of Nkx2.5+ cells in the myocardium of the different groups together with semi-quantitative analysis. Figures (a-f) represent: 9.5 weeks human fetal heart as a positive control (a), followed by left ventricular regions from the; control (b), pregnancy (c), myocardial infarction (d), ischemia-reperfusion (e) and ischemia-reperfusion+growth factors (f) groups, respectively. The delineated areas in figure b-f represent a higher magnification of that region. Bars in panel (a-f) = 100 µm. Figure (g) demonstrates the relative number of Nkx2.5+ cells in relation to the total number of cells in the different groups. Data is presented as mean± SD. *P<0.05. **Section B**: Representative stainings of c-Kit+cells in: adult rat kidney as positive control (a), followed by the left ventricular regions from the ischemia-reperfusion+growth factors (b, d) and myocardial infarction groups (e). The delineated area in figure (b) is magnified (100 x) in figure (c). In figures (b-e), red arrows indicate c-Kit+ cells. Bars in figures (a, b) = 100 µm and in figures (c-e) = 50 µm. Figure (f) demonstrates the percentage of c-Kit+cells in relation to the total number of cells in the different groups. Data is presented as mean ± SD. *P<0.05 and **P<0.001. **Section C**. Representative stainings of Isl1+ cells in whole heart cytospins from (c) pregnancy, (d) myocardial infarction, (e) ischemia-reperfusion and (f) ischemia-reperfusion+growth factors groups. Figures (a) and (b) represent the positive controls; whole rat embryo ED13 and insulin cell line-1E respectively. Delineated areas in figure c, d, e and f represent a higher magnification of that region. Bars in figures (a–f) = 100µm. In figure; ED: embryonic day; MI: myocardial infarction; IR: ischemia-reperfusion; IR+GF: ischemia-reperfusion+growth factors.

Blinded semi-quantitative IHC analyses of cells expressing c-Kit and Nkx2.5 were performed two weeks post-intervention. As demonstrated in figure 8A and B, the cellular protein expression of both Nkx2.5 and c-Kit follow the same pattern as previously shown both in Western blot and at the mRNA level, where the highest up-regulation was observed in the ischemia-reperfusion injury groups (IR and IR+GF).

In consistency with the low mRNA expression of Isl1 in the different groups, Isl1+ cells were extremely difficult to find in IHC sections of the hearts. In order to circumvent this problem, whole heart cytospins were performed where few Isl1+ cells could be identified in the different groups (Fig 8C). No reliable semi-quantitative analysis could be performed on the cytospinned material.

### Semi-quantitative Analysis of Mast Cell Expression

Mast cells were detected by Toluidine staining on whole heart cytospins at two weeks post-intervention. As shown in figure 7A and B, the positive mast cells stained red-purple and were easy to detect. The semi-quantitative analysis of mast cells, revealed a mismatch between the previously shown up-regulation of c-Kit (Fig 2A) and mast cells (Fig 7C). While pregnancy up-regulated c-Kit, mast cells were down-regulated. In the ischemia-reperfusion injury groups (IR and IR+GF) up-regulation of c-Kit (Fig 2A) was similar with and without growth factors, while addition of growth factors caused a much higher mast cell expression.

## Discussion

This is the first prospective and randomized study, which examines the time-dependent and spatial up-regulation of the best characterized cardiomyocyte progenitor markers c-Kit and Isl1, as well as the early cardiomyocyte marker Nkx2.5, during experimental pathological and physiological stress stimuli in adult rat hearts. Our study demonstrates global and focal up-regulations of c-Kit and Isl1, respectively, as well as Nkx2.5, after ischemia-reperfusion injury but also after myocardial infarction and pregnancy. Our findings are based on mRNA as well as protein analyses where both immunohistochemistry and Western blot analyses were performed on tissue specimens from the different regions of the heart. By using this strategy, the expressions of Isl1, c-Kit and Nkx2.5 were verified using three independent methods which strengthen our conclusions.

Ischemia-reperfusion injury induces the strongest up-regulation of c-Kit and Nkx2.5 expression, which occurs two weeks after the ischemic event and is found throughout the entire heart. There is also a spatial mismatch on one hand of c-Kit and Nkx2.5 and on the other Isl1 expression. Both c-Kit and Nkx2.5 are globally up-regulated, while the Isl1 up-regulation is localized to the outflow tract, where these cells previously have been demonstrated to reside in the rat and human embryonic hearts [17], [28].

The origin and phenotype of the c-Kit+ cells in the adult heart is still under debate. All c-Kit+ cells are not stem cells since c-Kit can also be expressed on both mast cells, residing in the myocardium [29] and on other hematopoietic cells [30], [31], [32], [33].

During myocardial ischemia cardiac mast cells play a pivotal role in the initiation of the inflammatory response by releasing pro-inflammatory mediators, which trigger a cytokine cascade [29], [34]. If c-Kit expression was solely due to mast cell accumulation and activation, the c-Kit expression would be correlated to changes in mast cell expression, which was not the case in this study. Beltrami and coworkers have previously demonstrated that there are small clusters of c-Kit+ cells in the interstitium between mature cardiomyocytes in rat hearts [10]. Furthermore, c-Kit+ cells have also been isolated from human hearts, and upon transplantation into infarcted myocardium of immunodeficient rats, they generated a chimeric heart, with new population of cardiomyocytes and vasculature [10], [35]. In keeping with Beltrami and associates we found that ischemia-reperfusion injury also seems to stimulate such a pool of “cardiogenic” c-Kit+ cells that give rise to early cardiomyocytes. Besides c-Kit, such cells also were reported to express the early cardiomyocyte marker Nkx2.5, a marker that is not expressed by mast cells [10]. How these cells get activated during ischemia-reperfusion is not known, but it might be related to the significantly increased endogenous production of both IGF-1 and HGF as seen in our study. Exogenous addition of IGF-1 and HGF could further boost the endogenous expression of these growth factors and further stimulate the expression of Nkx2.5, primarily in the remote left ventricle and peri-ischemic regions. These growth factors which are known to stimulate tissue regeneration [36], [37], [38], [39], might mediate up-regulation of stem cell factor (SCF), which has previously been shown to both stimulate proliferation and homing of c-Kit+ cells [10], [40]. Such mechanisms might explain the up-regulation of “cardiogenic” c-Kit+ cells during ischemia-reperfusion injury. This hypothesis is further supported by a recent study by Ellison and coworkers, where intra-coronary delivery of IGF-1/HGF significantly increased c-Kit+/Nkx2.5+ cardiac progenitor cells in the infarct and border regions in a pig ischemia model [41].

After myocardial infarction and compared to ischemia-reperfusion injury, there was a significantly lower accumulation of c-Kit+ cells in the heart, consistent with the lower expression of endogenous IGF-1 and HGF in the myocardial infarction group. It has been shown in other studies that myocardial infarction induces homing and proliferation of c-Kit+ cells to the border of the infarction area [10], [42], but following permanent coronary occlusion, such a homing effect may according to our results be less robust than that seen after ischemia-reperfusion injury.

The transcription factor Isl1 is expressed in the adult heart in the outflow tract [17] as confirmed in this study. Isl1 expression is stimulated only in the group exposed to ischemia-reperfusion injury and foremost after the addition of HGF and IGF-1. Isl1 is up-regulated primarily in the outflow tract but also in the left ventricle and the peri-ischemic region. Since the baseline expression is low it is unclear to what degree Isl1+ cells are contributing to the regeneration of new cardiomyocytes.

During pregnancy there is a general up-regulation of c-Kit and Nkx2.5, but not Isl1. This can be interpreted as the hormonal response to pregnancy stimulates cardiac stem cells and the generation of new cardiomyocytes, a phenomenon, which has not been described earlier. Previous studies have only implied that during pregnancy the heart adapts to an increased workload by left ventricle hypertrophy [43], [44]. According to our data, there also seems to be hyperplasia involved, through the up-regulation of cardiac progenitors. To some extent these c-Kit+ and Nkx2.5+ cells might originate from the fetus. This has been shown to be the case when pregnant mice are exposed to cardiac injury, where fetal cells home into the injured heart forming endothelial cells, smooth muscle cells and cardiomyocytes [45]. If these fetal cells will persist in the maternal tissue as microchimera to protect the maternal heart from cardiac injury even in the future needs to be explored.

In conclusion, ischemia-reperfusion injury is the strongest stimulus for activation of endogenous cardiomyocyte regeneration, correlating to the endogenous up-regulation of IGF-1 and HGF. These substances may become utilized to augment the endogenous regenerative capacity seen in patients with ischemic heart failure and thereby circumvent the need for stem cell transplantation.
